# The G Protein-Coupled Receptor (GPR) 15 Counteracts Antibody-Mediated Skin Inflammation

**DOI:** 10.3389/fimmu.2020.01858

**Published:** 2020-08-14

**Authors:** Lina Jegodzinski, Tanya Sezin, Karin Loser, Sadegh Mousavi, Detlef Zillikens, Christian D. Sadik

**Affiliations:** ^1^Department of Dermatology, Allergy, and Venereology, University of Lübeck, Lübeck, Germany; ^2^Department of Dermatology, University of Münster, Münster, Germany; ^3^Center for Research on Inflammation of the Skin, University of Lübeck, Lübeck, Germany

**Keywords:** GPR15, pemphigoid disease, epidermolysis bullosa acquisita, autoantibodies, skin inflammation, GPR15L

## Abstract

The G protein-coupled receptor 15 (GPR15) has recently been highlighted as an important regulator of T cell trafficking into the gut under physiological and pathophysiological conditions. Additionally, circumstantial evidence has accumulated that GPR15 may also play a role in the regulation of chronic inflammation. However, the (patho)physiological significance of GPR15 has, in general, remained rather enigmatic. In the present study, we have addressed the role of GPR15 in the effector phase of autoantibody-mediated skin inflammation, specifically in the antibody transfer mouse model of bullous pemphigoid-like epidermolysis bullosa acquisita (BP-like EBA). Subjecting *Gpr15*^−/−^ mice to this model, we have uncovered that GPR15 counteracts skin inflammation. Thus, disease was markedly aggravated in *Gpr15*^−/−^ mice, which was associated with an increased accumulation of γδ T cells in the dermis. Furthermore, GPR15L, the recently discovered cognate ligand of GPR15, was markedly upregulated in inflamed skin. Collectively, our results highlight GPR15 as counter-regulator of neutrophilic, antibody-mediated cutaneous inflammation. Enhancing the activity of GPR15 may therefore constitute a novel therapeutic principle in the treatment of pemphigoid diseases, such as BP-like EBA.

## Introduction

Recently, evidence has accumulated that the G protein-coupled receptor 15 (GPR15) may modulate chronic inflammation. GPR15 has been demonstrated, for instance, to be abundant on neutrophils and monocytes in the peripheral blood of rheumatoid arthritis (RA) patients as well as on macrophages and neutrophils in arthritic joints (1, 2). GPR15 is also highly expressed on a population of IL-17-producing CD4^+^ T cells emerging in the peripheral blood of patients with ulcerative colitis or multiple sclerosis (3, 4). It is also induced on T cells of cigarette smokers, a behavior leading to a state of chronic systemic low-level inflammation (1–4). However, the functional significance of the upregulation of GPR15 under these conditions has only been investigated in detail for the pathogenesis of gut inflammation.

Specifically, it has been demonstrated that, under homeostatic conditions, preferentially GPR15^+^ subpopulations of memory T cells and T_regs_ are recruited into the colon. GPR15 also mediates the recruitment of *ex vivo* polarized T_H_17 cells into the colon, collectively pointing at a role for GPR15 in T cell trafficking to the gut (5). The role of GPR15 has also been investigated in several mouse models of inflammatory bowel disease (IBD). This uncovered a complex, context-dependent role of GPR15 in colon inflammation: in support of a pivotal, disease-promoting role of GPR15 in colitis, GPR15 is required for T effector cell recruitment into the colon in the CD45RB^high^ T cell transfer model of colitis (6). Accordingly, GPR15 deficiency is protective in this model. In sharp contrast, colitis in the anti-CD40 antibody model is aggravated in *Gpr15*^−/−^ mice. These opposite net effects on colitis may be the result of fundamental differences in the actions of T_regs_ in the two models (6, 7).

Despite these multiple lines of evidence, suggesting a broader role of GPR15 in the pathogenesis of chronic inflammatory diseases, a potential role in antibody-mediated tissue inflammation has not been investigated. We therefore explored the role of GPR15 in the antibody transfer mouse model of bullous pemphigoid-like epidermolysis bullosa acquisita (BP-like EBA), a prototypical example for organ-specific, antibody-mediated autoimmunity (8, 9). BP-like EBA belongs to the group of pemphigoid diseases, a group of seven autoimmune diseases of the skin and mucous membranes featuring an immune response against well-defined autoantigens located in the dermal-epidermal adhesion complex (10).

In response to the deposition of autoantibodies, immune cells, particularly granulocytes are recruited to the dermal-epidermal junction (DEJ), where they subsequently degrade the dermal-epidermal adhesion complex, thus, compromising dermal-epidermal adhesion and eliciting the formation of subepidermal clefts, which clinically appear as tense skin blisters and erosions (8). The mechanisms choreographing the recruitment and activity of granulocytes in the dermis are only partially understood. Recent studies in mouse models of pemphigoid diseases, have highlighted the anaphylatoxin C5a and the eicosanoid leukotriene B_4_ as central choreographers of this process on the molecular level (9, 11, 12). On the cellular level, γδ T cells and T_regs_ have been demonstrated to interact with granulocytes to promote and suppress skin inflammation, respectively, by modulating neutrophil activity in the dermis (13–15).

In the present study, we employed the previously described *B6;129P2-Gpr15*^*tm*1.1*Litt*^*/J* mouse strain, in which the *Gpr15* gene is replaced by a gene sequence encoding green fluorescent protein (GFP) (7). Homozygous *B6;129P2-Gpr15*^*tm*1.1*Litt*^*/J* mice (*Gpr15*^*Gfp*/*Gfp*^; *Gpr15*^−/−^ mice) do not express GPR15 but instead GFP when *Gpr15* is transcribed. The *B6;129P2-Gpr15*^*tm*1.1*Litt*^*/J* strain can therefore be utilized as both knockout and reporter line.

Subjecting *Gpr15*^−/−^ mice to the antibody transfer BP-like EBA model, we found disease in these mice markedly aggravated compared to wild-type littermate controls, hence, indicating a protective role of GPR15 in antibody-mediated skin inflammation. This aggravation of skin inflammation was associated with a pronounced induction of the recently identified cognate ligand of GPR15, GPR15L (16, 17), and an increased accumulation of γδ T cells in the dermis. With γδ T cells previously demonstrated to aggravate disease in the BP-like EBA model (13), we conclude that GPR15 may mediate partial protection from antibody-mediated skin inflammation by limiting the recruitment of γδ T cells into the dermis.

## Results

### GPR15 Deficiency Aggravates Antibody Transfer BP-Like EBA

**Figure 1:**
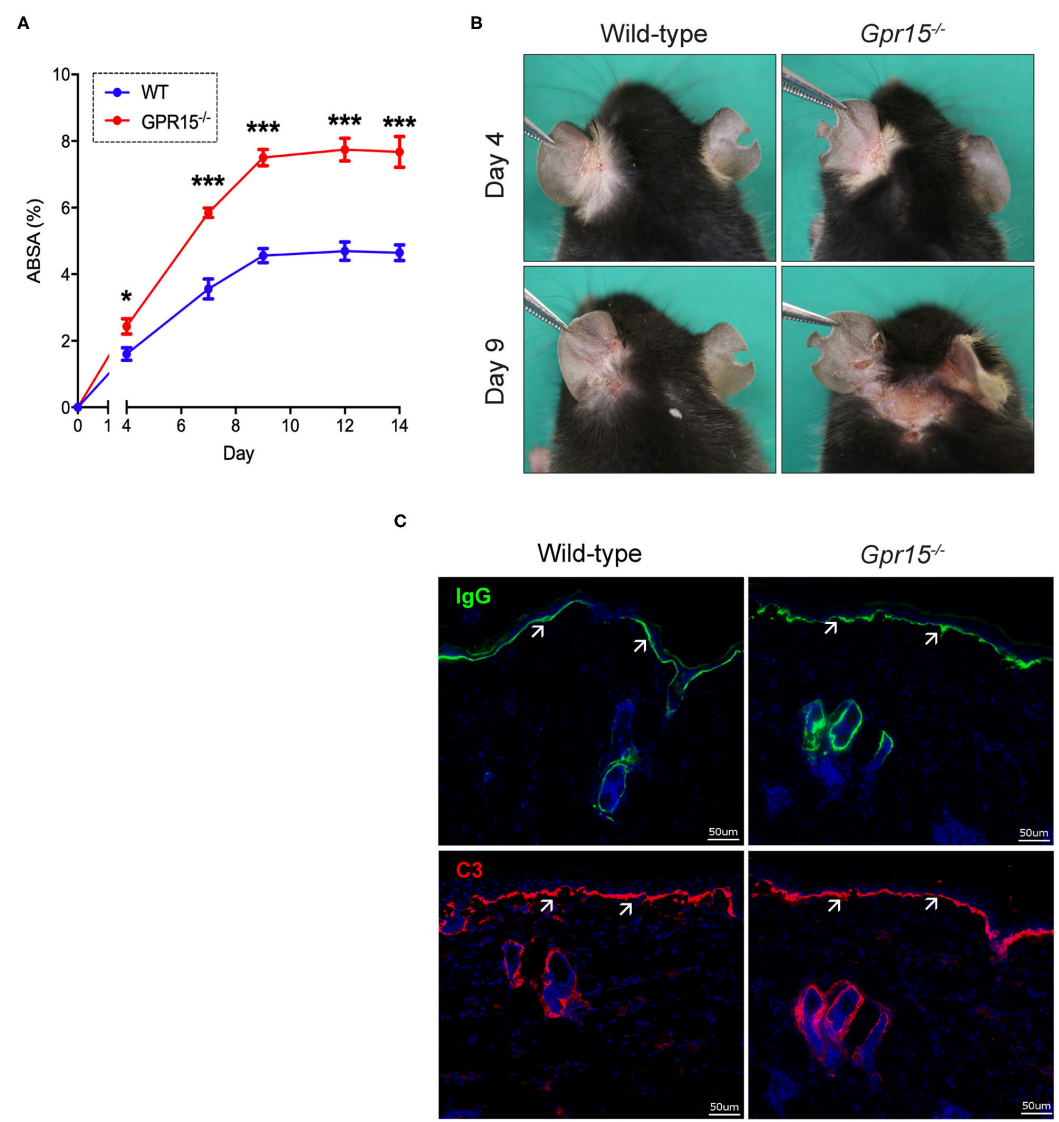
GPR15 counteracts BP-like EBA. The course of skin inflammation in the BP-like EBA mouse model was contrasted in wild-type and *Gpr15*^−/−^ mice. **(A)** Progression of disease severity benchmarked as percentage of the total body surface affected by skin lesions (ABSA) over the course of 14 days. **(B)** Representative pictures of the clinical presentation of wild-type and *Gpr15*^−/−^ mice on days 4 and 9 of the experiment. **(C)** Direct immunofluorescence microscopy of perilesional skin for IgG (upper panel) and C3 (lower panel) with white arrows indicating linear depositions of IgG and C3, respectively. Results are presented as mean ± SEM of the ABSA (*n* = 10 mice/group; pooled from three independent experiments). Results were analyzed by two-way ANOVA and Holm-Sidak's multiple comparison test. *p < 0.05 and ***p < 0.001 for the comparison between wild-type *vs. Gpr15*^−/−^ mice on the day indicated.

Wild-type and *Gpr15*^−/−^ mice were subjected to the antibody transfer BP-like EBA mouse model and the course of disease was monitored for 14 days. Both groups started to develop signs of skin inflammation within 4 days upon the first administration of anti-COL7c IgG (Figures 1A,B). Disease severity in both groups further increased and plateaued around day 8. Disease was, however, significantly more severe in *Gpr15*^−/−^ mice throughout the entire time of observation (Figure 1A). Specifically, when disease had plateaued in both groups, skin inflammation in *Gpr15*^−/−^ mice affected approximately twice more of the total body surface than in wild-type mice. By immunofluorescence staining of perilesional skin of wild-type and *Gpr15*^−/−^ mice for IgG and C3, we subsequently demonstrated that both IgG and C3 were deposited at the dermal-epidermal junction (DEJ) in both groups, thus, confirming the specificity of skin inflammation observed in our experiment (Figure 1C).

### Subepidermal Cleft Formation and Dermal Accumulation of γδ T Cells Are Increased in *Gpr15^−/−^* Mice

**Figure 2:**
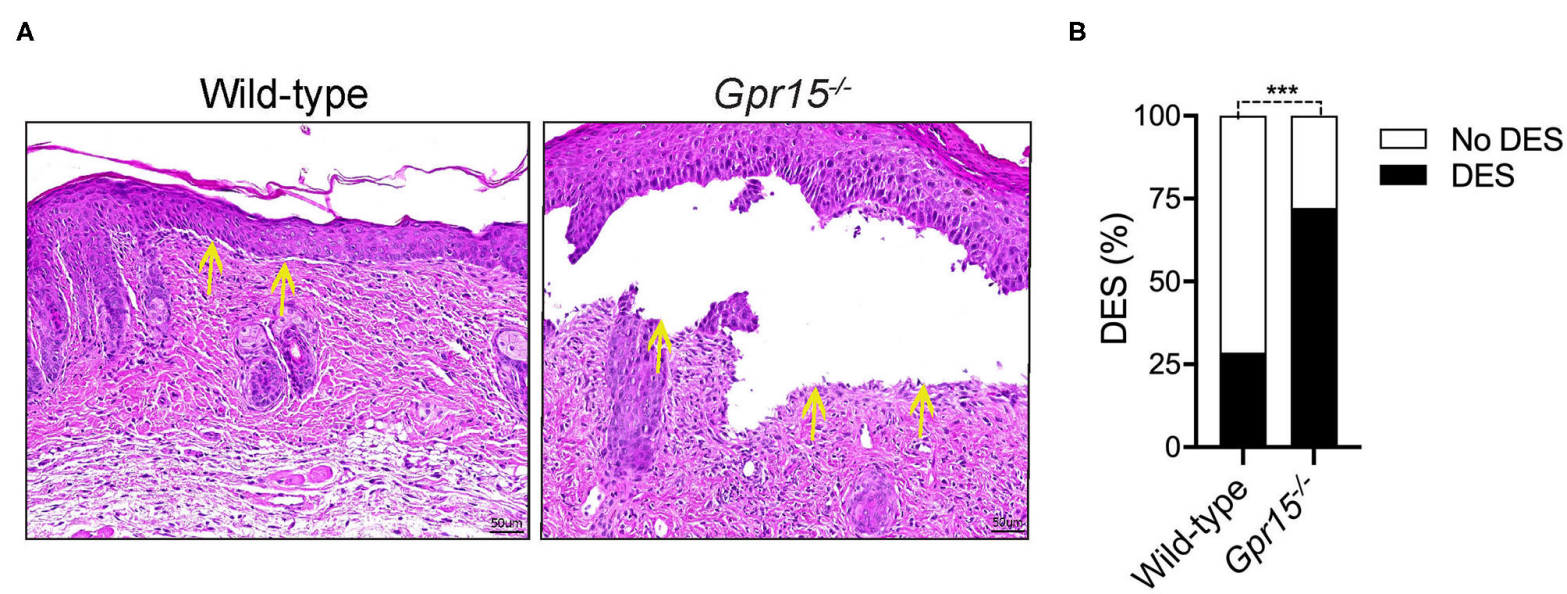
GPR15 deficiency exacerbates subepidermal cleft formation. Histopathological analysis of skin harvested on day 14 in the BP-like EBA model. **(A)** Representative pictures of H&E stainings of lesional skin. Yellow arrows indicate sites of dermal-epidermal clefts. **(B)** Frequency of dermal-epidermal separation (DES) in skin lesions in wild-type and *Gpr15*^−/−^ mice (*n* = 6–7 mice per group; pooled from three independent experiments). Results were compared by Fisher's exact test. ***p < 0.001.

Lesional skin of both wild-type and *Gpr15*^−/−^ mice exhibited a marked inflammatory infiltrate of the dermis as well as subepidermal clefts, the histopathological correlate of blisters and erosions in BP-like EBA, at the end of the experiment on day 14 (Figure 2A). Quantifying the extent of subepidermal cleft formation revealed that it was significantly more pronounced in the *Gpr15*^−/−^ group (Figure 2B), suggesting a higher activity of neutrophils, the major drivers of cleft formation, in the dermis of *Gpr15*^−/−^ mice.

**Figure 3:**
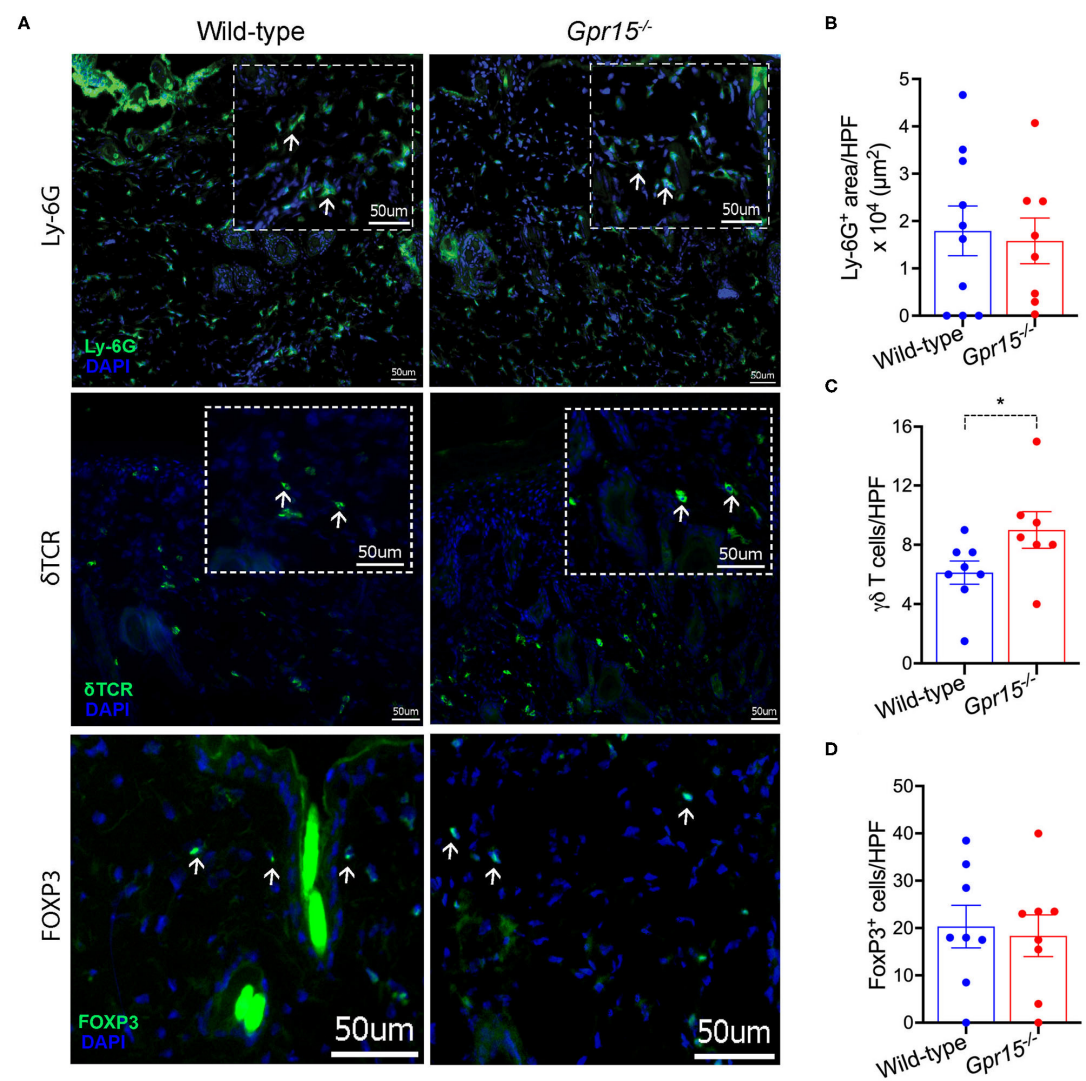
Quantification of cellular infiltration of perilesional skin. Infiltration of perilesional skin by Ly-6G^+^, δTCR^+^, and FOXP3^+^ cells on day 14 of the BP-like EBA mouse model was evaluated. **(A)** Representative pictures of stainings for Ly-6G^+^ (upper panel), δTCR^+^ (middle panel), and FOXP3^+^ cells (lower panel). Inserts in dashed lines represent 3× digital magnifications of selected areas of the dermis. White arrows indicate examples for positively stained cells. Scale bar represents 50 μm. Quantification of **(B)** Ly-6G^+^ cells **(C)** δTCR^+^, and **(D)** FOXP3^+^ cells. Results are presented as mean ± SEM (*n* = 7–10 mice per group, pooled from three independent experiments) and were compared by Mann-Whitney test. *p < 0.05.

We next determined the density of neutrophils (Ly-6G^+^), γδ T cells (γδ TCR^+^), and regulatory T cells (T_regs_; FOXP3^+^), which are the three cell populations previously implicated in the regulation of skin inflammation in the BP-like EBA model, in perilesional skin by immunofluorescence stainings. Neutrophils were abundant in the dermis of both wild-type and *Gpr15*^−/−^ mice (Figure 3A), and their numbers did not differ between the two groups in our experiments (Figure 3B). Also, in both groups, significant numbers of γδ T and T_reg_ cells infiltrated perilesional skin (Figure 3A). While we did not find a difference in the number of T_regs_ between the two groups (Figure 3D), γδ T cells were significantly more abundant in the dermis of *Gpr15*^−/−^ mice than in wild-type mice (Figure 3C).

### Expression of GPR15 and GPR15L in BP-Like EBA

**Figure 4:**
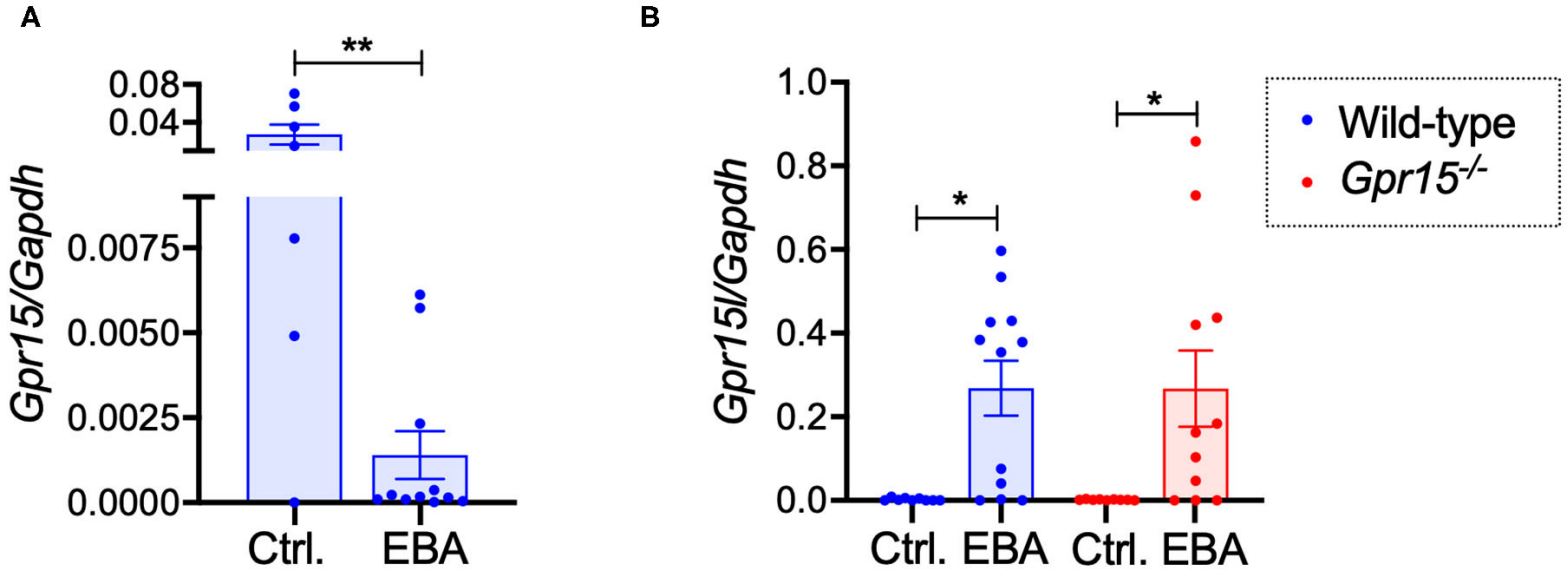
GPR15 and GPR15L mRNA expression in perilesional skin. mRNA expression levels were determined in naïve skin of healthy mice and of perilesional skin of mice suffering from BP-like EBA on day 14 of the experiment. **(A)** GPR15 mRNA levels in wild-type mice. **(B)** GPR15L mRNA levels in wild-type and *Gpr15*^−/−^ mice. Results are presented as mean ± SEM (*n* = 6–12 mice per group, pooled from three independent experiments) and were compared in **(A)** by Mann-Whitney test and in **(B)** by Kruskal-Wallis test and Dunnett's multiple comparison test. *p < 0.05; **p < 0.01.

Assessing the expression levels of GPR15 and its ligand GPR15L on mRNA level in naïve control skin and in perilesional skin harvested on day 14 revealed that GPR15 and GPR15L were reversely regulated. While GPR15 mRNA was expressed in naïve wild-type skin on relatively high levels, its expression was significantly lower in inflamed skin (Figure 4A). GPR15L, in contrast, was barely detectable in naïve skin but was markedly upregulated in inflamed skin (Figure 4B). There was no difference in the expression levels of GPR15L in wild-type and *Gpr15*^−/−^ mice (Figure 4B).

**Figure 5:**
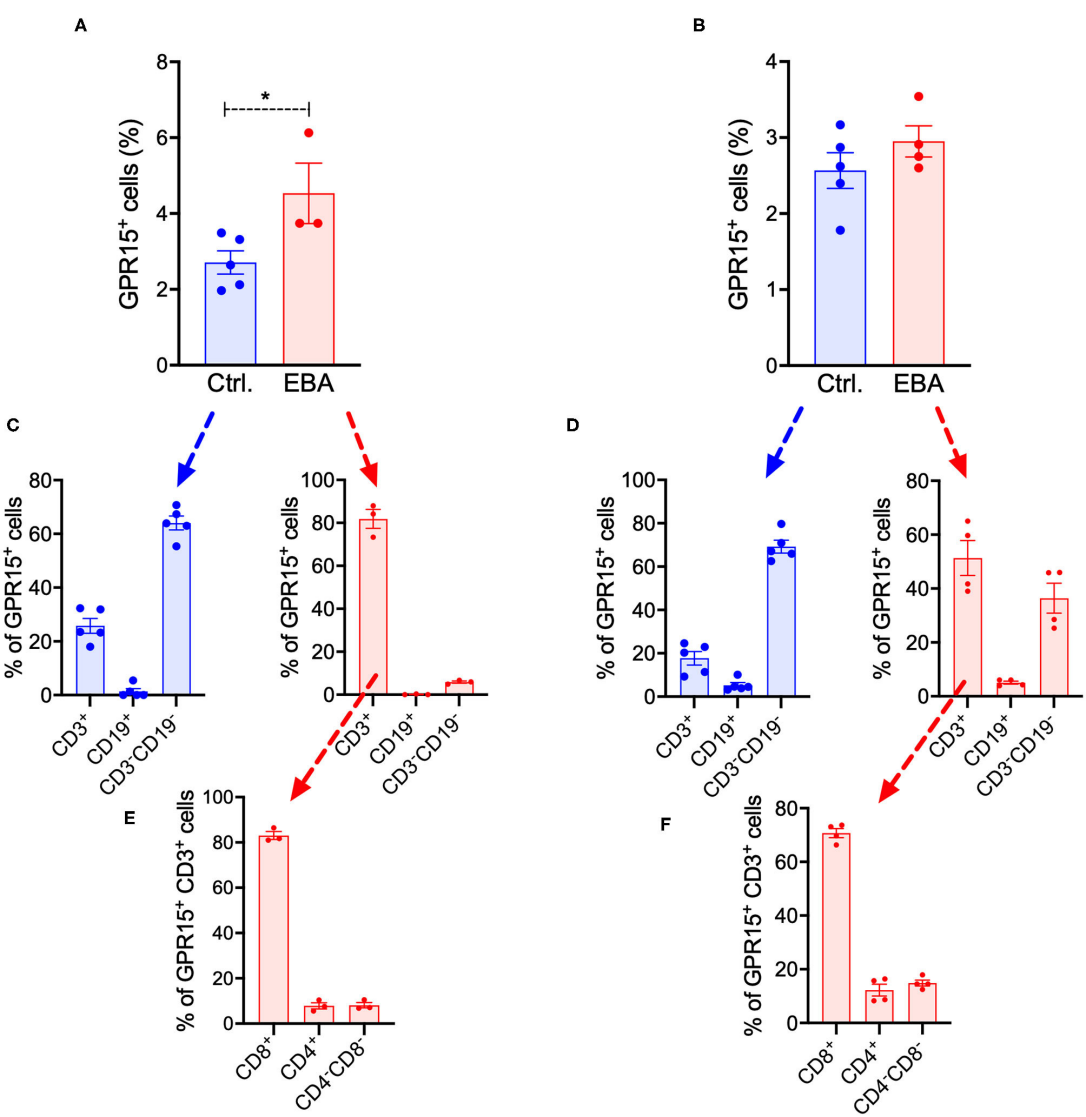
Systemic expression of GFP in BP-like EBA. Quantification of GFP^+^ cells in **(A)** inguinal lymph nodes and **(B)** the spleen of control (Ctrl.) and diseased *Gpr15*^−/−^ mice on day 14 following induction of BP-like EBA as assessed by flow cytometry. Relative distribution of GFP^+^ cells among T (CD3^+^) cells, B (CD19^+^) cells, and non-lymphocyte cells (CD3^−^CD19^−^) in control and diseased *Gpr15*^−/−^ mice on day 14 of the experiment in **(C)** inguinal lymph nodes and **(D)** the spleen. Relative distribution of GFP^+^ cells among CD4^+^ and CD8^+^ T cells in control and diseased *Gpr15*^−/−^ mice on day 14 of the experiment in **(E)** lymph nodes and **(F)** the spleen. Results are presented as mean ± SEM (*n* = 3–4 mice per group). Results in **(A,B)** were compared by Mann-Whitney test. *p < 0.05.

We also assessed the frequency of GPR15^+^ cell populations in inguinal lymph nodes (LNs) and in the spleen of *Gpr15*^−/−^ *(Gpr15*^*Gfp*/*Gfp*^*)* mice under naïve conditions and in EBA on day 14 (Figures 5A,B). The complete gating strategy of these experiments is summarized in Supplementary Figures 1, 2. Under naïve conditions, in both LNs and spleen, ~2–3% of living cells expressed GPR15 (Figures 5A,B). This percentage was slightly increased in the LNs in EBA (Figure 5A). To characterize the cell populations expressing GPR15, we stained for the T cell marker CD3 and the B cell marker CD19 (Figures 5C,D). While under naive conditions GPR15^+^ cells were mainly CD3^−^CD19^−^, in EBA a significant proportion of GPR15^+^ cells expressed CD3. All along there was no co-expression of GPR15 and CD19 (Figures 5C,D). To further differentiate the GPR15^+^CD3^+^ cell population on day 14 of the experiment, we determined their expression of CD8 and CD4 (Figures 5E,F). GPR15 was mostly expressed on CD8^+^ cells. However, there was no difference between the number of CD8^+^CD3^+^ cells in the lesional skin of wild-type and *Gpr15*^−/−^ mice (Supplementary Figure 3). Wild-type and *Gpr15*^−/−^ mice did not differ in the number of T_regs_ and γδ T cells in the lymph nodes and the spleen (Supplementary Figure 4). There was no co-expression between GFP (GPR15) and the neutrophil Ly-6G/CD11b (Supplementary Figure 5).

## Discussion

In the present study, we have uncovered that GPR15 plays a significant, protective role in the effector phase of BP-like EBA. The pronounced aggravation of skin inflammation in *Gpr15*^−/−^ mice at the clinical level was paralleled at the histopathological level by an enhancement in the formation of subepidermal clefts. The latter emerge through the actions of neutrophils, which includes the release of proteases and of reactive oxygen species, degrading the dermal-epidermal adhesion complex (8). The increase in subepidermal cleft formation is, consequently, a sign of enhanced neutrophil activity in the skin. The activity of neutrophils in the BP-like EBA model is modulated by T_regs_ and γδ T cells, which inhibit and promote, respectively, the response of neutrophils to immune complexes by interacting with neutrophils within the dermal infiltrate (13, 15). Our finding that γδ T cells were more abundant in the dermal infiltrate of *Gpr15*^−/−^ mice is, therefore, well in line with these previously published results and suggests that a direct or indirect effect of GPR15 on the recruitment of γδ T cells, and the subsequent activating effect of the latter on neutrophils may be a major mechanism of the aggravation of skin inflammation in *Gpr15*^−/−^ mice.

Although we detected GPR15 in the skin on mRNA level, we did not find GPR15 on protein level in the skin. There are several plausible explanations for this constellation: first, we only examined GPR15 protein expression on day 14 of the experiment. The receptor may be active in the skin at an earlier stage of the pathogenesis of diseases and may already inactive at the stage of full-blown skin inflammation on day 14. Second, GPR15 may modulate tissue inflammation from outside the skin. Third, like many other GPCRs, GPR15 may be internalized and degraded after binding of its ligand GPR15L in the skin.

The contribution of processes outside the skin to the BP-like EBA mouse model are largely unknown and disease is, accordingly, supposed to be predominantly driven by processes exclusively proceeding in the skin. Furthermore, depletion of CD8^+^ cells did alter the course of BP-like EBA (15), and there was not difference in the number of CD8^+^CD3^+^ cells in lesional skin in wild-type and *Gpr15*^−/−^ mice, which collectively argues against a significant contribution of GPR15^+^CD8^+^ cells increasing in BP-like EBA in the spleen and lymph nodes.

Supporting local actions of GPR15, its cognate ligand GPR15L was markedly upregulated in the skin. However, GPR15 mRNA levels were, in contrast, reduced in inflamed skin. A potential explanation for this situation is that GPR15L/GPR15 may exert inhibitory or chemo-repulsive effects on the migration of GPR15^+^ cells, thus, counteracting the recruitment of certain cell populations, including γδ T cells, into the dermis. Such a mode of action of GPR15L/GPR15 would also explain why we did not find GFP expression in the skin of *Gpr15*^−/−^ (*Gpr15*^*Gfp*/*Gfp*^) mice but in their lymph nodes, spleens, and peripheral blood. It would be also another plausible explanation why we did not detect GPR15 on protein level in the skin. In line with this notion, the structure of GPR15 resembles that of CC chemokine receptors but exhibits unique peculiarities (17), and it is still contended whether GPR15L can induce migration of GPR15^+^ cells. Thus, although one study reported that GRP15L chemoattracts T cells (16), another study did not find GPR15L to chemoattract T cells but to dose-dependently inhibit their chemoattraction toward CXCL12 (17).

Collectively, our results demonstrate that GPR15 is a regulator of tissue inflammation outside the colon and beyond immune responses predominantly driven by T cells. This finding is of importance as upregulation of GPR15 expression has been reported for a growing number of chronic inflammatory conditions (1–4), but its significance has remained largely elusive. As GPR15 exerted protective effects in BP-like EBA, its activation may be effective as novel therapeutic principle in the treatment of this disease and, possibly, other pemphigoid diseases. It is, therefore, intriguing that GPR15L, which is also known as AP-57 or C10orf99 and exhibits antimicrobial activities, can be applied in an *in situ* gel-forming hydrogel system (AP-57-NPs-H) onto the skin (18, 19), thus, making clinical studies examining the therapeutic effectivity of GPP15 activation in pemphigoid diseases in principle possible.

## Materials and Methods

### Mice and Genotyping

Previously described *129P2-Gpr15*^*tm*1.1*Litt*/*J*^ mice (*Gpr15*^−/−^ mice) on the *C57BL/6J* background were purchased from *The Jackson Laboratory* (Bar Harbor, ME, USA) (7). All experiments were conducted with *Gpr15*^−/−^ (*Gpr15*^*Gfp*/*Gfp*^) mice and their wild-type littermates in the age of 8–16 weeks. Mice were bred in the animal facility of the University of Lübeck (Lübeck, Schleswig-Holstein, Germany). All animal experiments had been approved by the local government. The mice were genotyped, as previously described (20).

### Antibody Transfer Bullous Pemphigoid-Like Epidermolysis Bullosa Acquisita (BP-Like EBA) Mouse Model

To generate anti-Col7 IgG, white New Zealand rabbits were immunized against the C epitope of type VII collagen. Purified anti-Col7 IgG were filter-sterilized (pore size 0.2 μm), quantified by NanoDrop (Thermo Fischer Scientific GmbH, Dreieich, Germany), and assessed for their reactivity to murine Col7 by indirect immunofluorescence analysis performed on murine tail skin sections as previously described (21). Antibody transfer BP-like EBA was induced, as previously described (12, 21). Briefly, mice were injected s.c. with 50 μg of affinity purified anti-Col7 IgG on days 0, 2, and 4 of the experiment. To score the severity of disease, skin areas exhibiting erythema, blisters, erosions, crusts, or alopecia were categorized as “affected.” Subsequently, the percentage of the total body surface affected by skin lesions (ABSA) was calculated on the days indicated in the figures. On day 14, mice were euthanized, and tissue specimens were harvested.

### Histopathology

For histopathology, skin biopsies of lesional skin were fixed in 4% (w/v) buffered formalin (Carl Roth, Karlsruhe, Germany) and 6-μm sections from paraffin-embedded tissues were stained with hematoxylin and eosin (H&E). The extent of dermal-epidermal separation (DES) was assessed by individually categorizing three sections per animal with “0” if no DES was evident and “1” if DES was evident. The frequency of positive images for DES out of total number of images vs. the frequency of negative images for DES out of total number of images was compared using the Fisher's test, as previously described (12).

### Immunofluorescence and Immunohistochemistry Stainings

To detect IgG and C3 depositions in perilesional skin, direct immunofluorescence microscopy was performed. Briefly, Alexa Fluor® 594 AffiniPure donkey anti-rabbit IgG (Jackson Immunoresearch, Suffolk, UK) and purified rat anti-mouse complement C3 IgG (CADARLANE, Ontario, Canada) were used to stain IgG and C3.

To quantify the extent of infiltration of perilesional skin with individual immune cells lineages, immunohistochemistry stainings of 6-μm skin cryosections were performed. The primary and secondary antibodies used in these efforts are compiled in Supplementary Table 1. Snap frozen cryosections were fixed in cold acetone for 10 min at −20°C, blocked, and incubated with the respective primary and secondary antibodies. Finally, slides were mounted with DAPI fluoromount G (SouthernBiotech, Birmingham, AL, USA).

Both IF and IHC stainings were visualized and photographed on the BZ-9000E series Keyence microscope (Keyence GmbH, Neu-Isenburg, Germany). Images were analyzed using the BZ-II Analyzer software (Keyence GmbH, Neu-Isenburg, Germany). For better illustration, image taken in 200× magnification were digitally further 3-fold magnified using the BZ-II Analyzer software where indicated in the manuscript. To quantify skin infiltration with neutrophils (Ly-6G^+^), the hybrid cell count function on BZ-II Analyzer software was used; to quantify infiltration by T cells and their subsets (FOXP3^+^, γδ TCR), counts of positively stained were manually determined in two independent 200× magnification fields per mouse and averaged.

### RNA Isolation and qPCR

Total RNA was extracted from snapped frozen perilesional skin samples using TRIzol™ reagent (Thermo Fischer Scientific GmbH, Dreieich, Germany) following the manufacturer's instructions. RNA concentrations were determined by a Nanodrop 2000c spectrophotometer (Thermo Fischer Scientific GmbH, Dreieich, Germany). 500 ng of total RNA were transcribed using the ReverseAid First Strand cDNA Synthesis Kit (Thermo Fischer Scientific GmbH, Dreieich, Germany). qPCR was performed using cDNA, diluted 1:10 in water, and the SYBR Select Master Mix (Thermo Fischer Scientific GmbH, Dreieich, Germany) according to the manufacturer's instructions. All primers used in this study were purchased from biomers.net (biomers.net GmbH, Ulm, Germany). The sequences used were for Gapdh forward primer 5′-AGGTCGGTGTGAACGGATTTG-3′ and reverse primer 5′-TGTAGACCATGTAGTTGAGGTCA-3′, for Gpr15 forward primer 5′-CGTTATTATTGCGGTGGCGG-3′ and reverse primer 5′-TCTGGCTGGAACCCTGAAAC-3′ and for Gpr15l forward primer 5′-CACCACCCATGACTTGACTG-3′ and reverse primer 5′-CTTCTAGCCCTTTCCGGTCT-3′. qPCR was performed on the Eppendorf Mastercycler ep Realplex (Eppendorf, Hamburg, Germany) with the following cycling conditions: 50°C for 2 min, 95°C for 2 min, followed by 40 cycles each of 95°C for 15 s, and 60°C for 1 min each. The expression level of the gene of interest was normalized to the mRNA expression level of *Gapdh*.

### Flow Cytometry

Organs were freshly harvested and meshed on a 70 μm filter. Thereafter, samples were lysed with erythrocyte lysis buffer (Qiagen GmbH), washed twice with FACS buffer (3% BSA in 0.01 M PBS pH 7.2) and blocked with FcR block (Miltenyi Biotec) prior to staining. Subsequently, cells were incubated for 30 min at 4°C with the following antibodies: 3 μg/mL PE-Vio770 conjugated anti-CD3 (REA641, Miltenyi Biotec), 2 μg/mL PE anti-CD8a (53-6.7, eBioscience), 2 μg/mL PerCP anti-CD4 (RM4-5, BD Biosciences), and 3 μg/mL APC-Vio770 anti-CD19 (6D5, Miltenyi Biotec) antibodies. 1:1,000 (v/v) DAPI (SouthernBiotech) was used for discrimination of dead cells. Samples were acquired on a MACSQuant flow cytometer and analyzed with FlowJo software V10.

### Statistical Analyses

All analyses were performed on raw data using GraphPad Prism 8.3 (GraphPad, San Diego, CA, USA). Results were compared by Mann-Whitney test, Fisher's exact test, two-way ANOVA with Holm-Sidak's multiple comparison test, or Kruskal-Wallis test with Dunnett's multiple comparison test, as detailed in the figure legends. *p* < 0.05 was considered statistically significant throughout the study.

## Data Availability Statement

The raw data supporting the conclusions of this article will be made available by the authors, without undue reservation, to any qualified researcher.

## Ethics Statement

The animal study was reviewed and approved by Animal Protection Committee of the state of Schleswig-Holstein.

## Author Contributions

CS, TS, and LJ planned the study, analyzed the results, and wrote the paper. DZ and KL analyzed the results and edited the paper. TS, LJ, and SM conducted the experiments. All authors contributed to the article and approved the submitted version.

## Conflict of Interest

The authors declare that the research was conducted in the absence of any commercial or financial relationships that could be construed as a potential conflict of interest. The handling editor declared a past co-authorship with several of the authors TS, SM, DZ, and CS.
