# Supplementary figures and images for: The G Protein-Coupled Receptor (GPR) 15 Counteracts Antibody-Mediated Skin Inflammation
Source: Front Immunol. 2020 Aug 14;11:1858. doi: 10.3389/fimmu.2020.01858 (PMC7456807; doi:10.3389/fimmu.2020.01858)

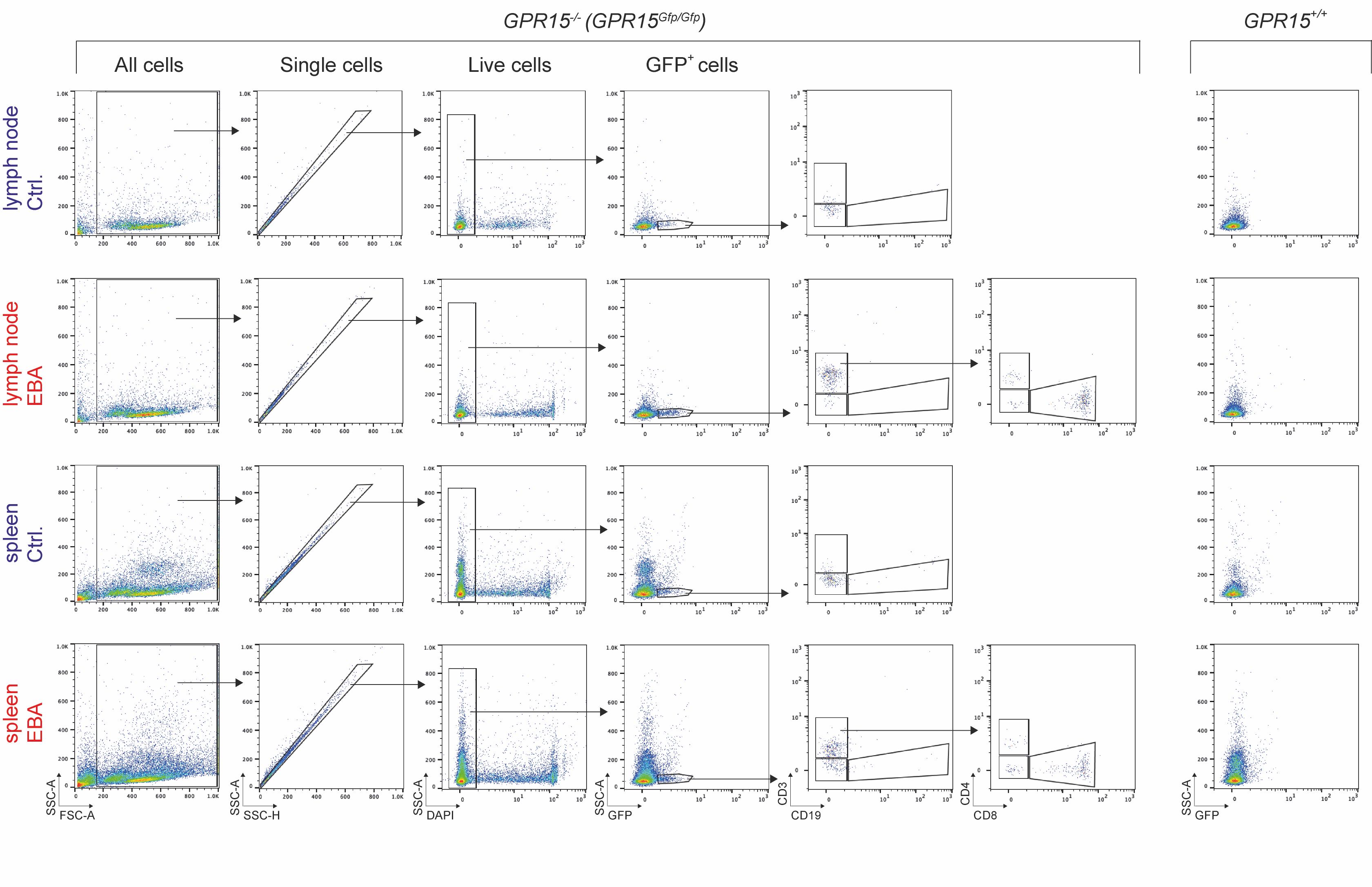

Supplement: Supplementary file 1 [file Image_1.JPEG]

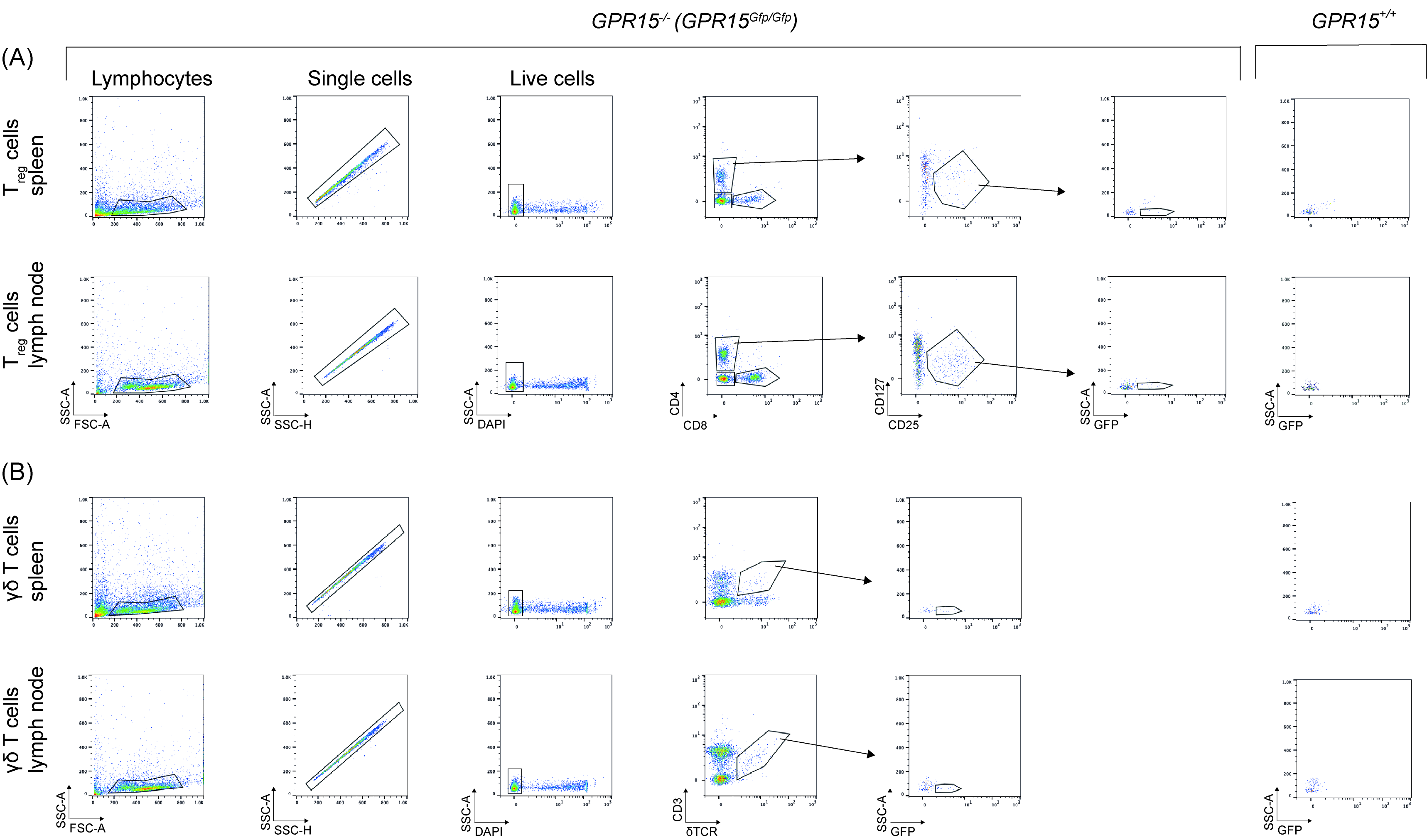

Supplement: Supplementary file 2 [file Image_2.TIF]

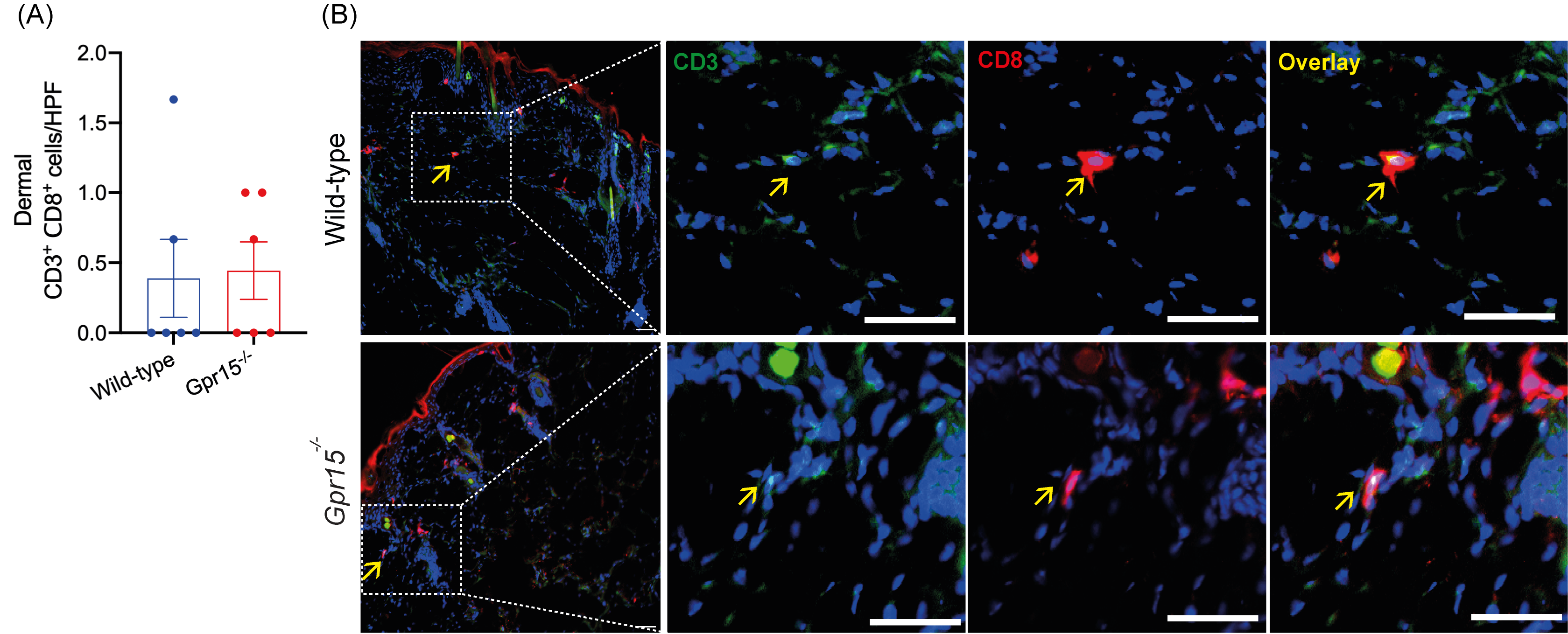

Supplement: Supplementary file 3 [file Image_3.TIFF]

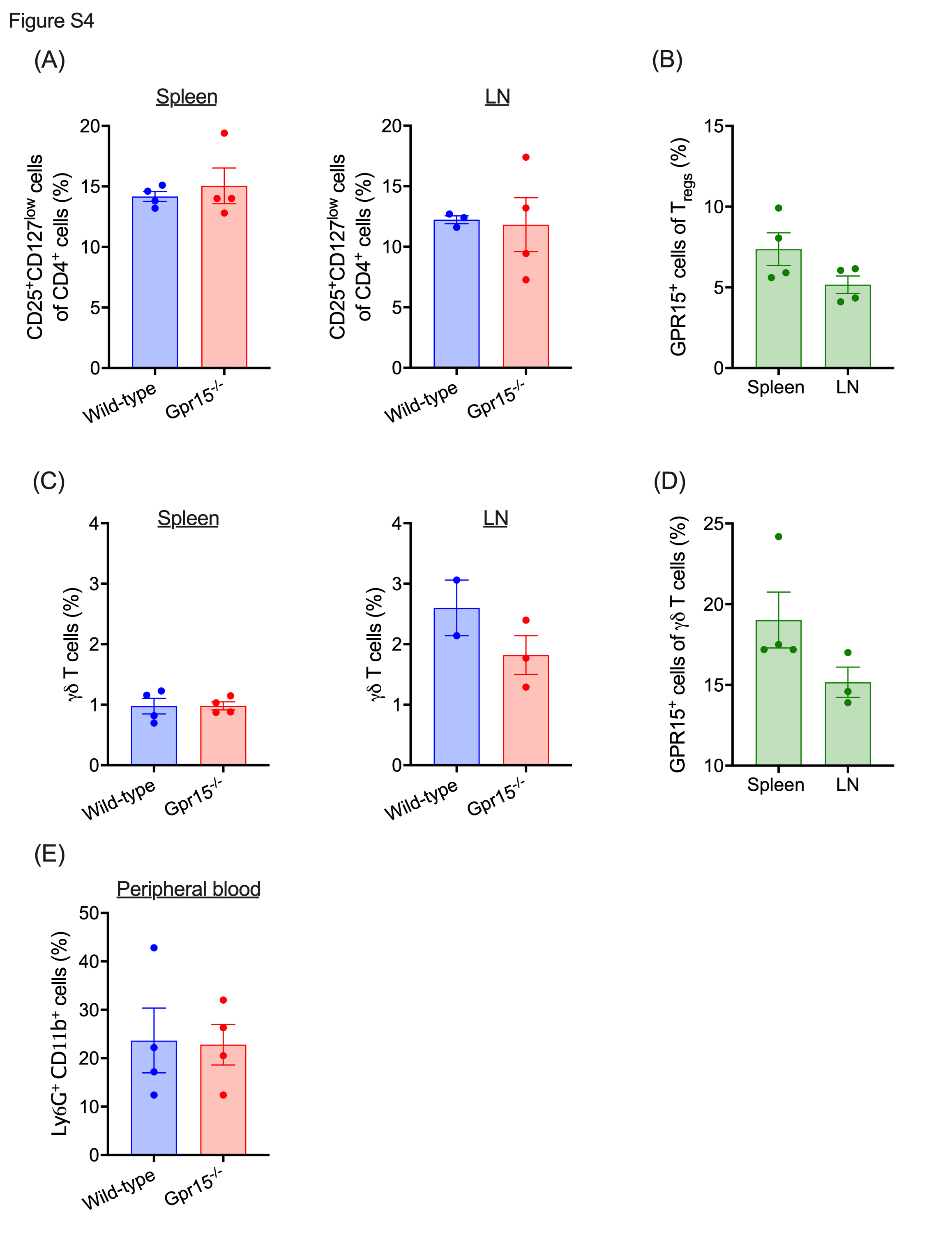

Supplement: Supplementary file 4 [file Image_4.TIFF]

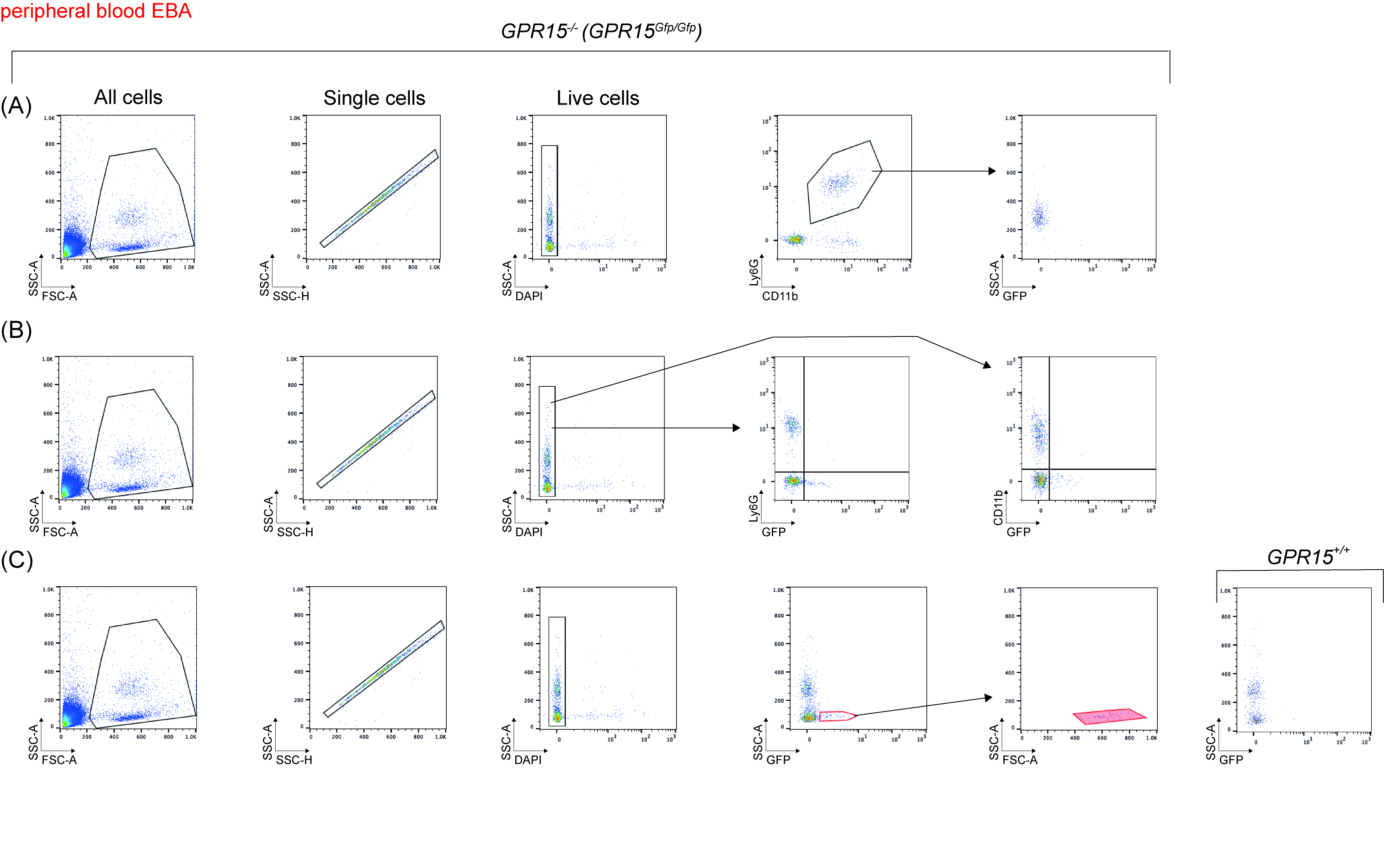

Supplement: Supplementary file 5 [file Image_5.TIF]
