# Supplementary material for: The G Protein-Coupled Receptor (GPR) 15 Counteracts Antibody-Mediated Skin Inflammation
Source: Front Immunol. 2020 Aug 14;11:1858. doi: 10.3389/fimmu.2020.01858 (PMC7456807; doi:10.3389/fimmu.2020.01858)
Supplement: Supplementary file 7 [file Table_2.docx]

**Supplement figure 1. Gating strategy for GFP detection and analysis.** Our gating strategy for GFP expression in lymph nodes and spleen of *Gpr15^-/-^ (Gpr15^gfp/gfp^)* mice before and after induction of BP-like EBA (day 14) is illustrated. Subsequent analysis of CD3 and CD19 expression of the GFP^+^ population is illustrated as well as the distribution of the main T cell subpopulations on day 14. *GPR15^+/+^* mice are used as negative control.

**Supplement figure 2. Gating strategy for T_regs_ and** $\boldsymbol{\gamma\delta}$**T cells in spleen and lymph nodes.**

(**A**) Gating of lymphocytes, single cells, viable cells for CD4^+^CD25^+^CD127^low^ cells and their GFP expression is shown for spleen and lymph node. (**B**) Gating of lymphocytes, single cells, viable cells for CD3^+^ $\delta$TCR^+^ cells and their GFP expression is illustrated for spleen and lymph node. *Gpr15^+/+^* mice are used as negative control.

**Supplement figure 3. Quantification of CD8^+^ T cell infiltration of perilesional skin.** Infiltration of perilesional skin by CD8^+^ T cells on day 14 of the BP-like EBA mouse model was evaluated. (**A**) Quantification of CD8^+^CD3^+^ cells. (**B**) Representative pictures of the stainings for CD3 and CD8. Inserts in dashed lines show 3x digital magnifications of the selected areas of the dermis indicated. Yellow arrows indicate examples for double-positively stained cells. Scale bar represents 50 µm. Results are presented as mean ± SEM (n = 6 per group, pooled from three independent experiments) and were compared by Mann-Whitney test.

**Supplement figure 4. GFP expression on T_regs_,** $\boldsymbol{\gamma\delta}$**T cells, and neutrophils.** (**A**) Quantification of T_regs_ in spleen and inguinal lymph nodes (LN) of diseased wild-type and *Gpr15^-/-^* mice on day 14 following induction of BP-like EBA as assessed by flow cytometry. (**B**) Percentages of GFP^+^ cells out of all T_regs_ in spleen and lymph node. (**C**) $\gamma\delta$T cells in spleen and lymph nodes of diseased wild-type and *Gpr15^-/-^* mice. (**D**) Fractions of GFP^+^ cells out of all $\gamma\delta$T cells in spleen and lymph node. (**E**) Quantification of Neutrophils in peripheral blood of diseased wild-type and *Gpr15^-/-^* mice. Results are presented as mean ± SEM (n = 2 – 4 mice per group). Results were compared by Mann-Whitney test.

**Supplement figure 5. GFP is not expressed on neutrophils in the peripheral blood.** (**A**) Gating of all cells, single cells, viable cells for Ly6G^+^ CD11b^+^ neutrophils and their GFP expression in peripheral blood of *Gpr15^-/-^* mice after induction of BP-like EBA (day 14) is shown. (**B**) Staining for Ly6G^+^CD11b^+^GFP^+^ cells. (**C**) Detection of the location of GFP^+^ cells in the SSC-A/FSC-A scatter is marked in red color. *GPR15^+/+^* mice were used as negative control.
